# Crystallization of stardust analogs under an electron flux atmosphere

**DOI:** 10.1093/pnasnexus/pgag095

**Published:** 2026-04-04

**Authors:** Rakibul A Shohan, Cody Cly, Angela Speck, Benjamin Sargent, Joseph A Nuth, Alan Whittington, Arturo Ponce

**Affiliations:** Department of Physics and Astronomy, University of Texas at San Antonio, San Antonio, TX 78249, USA; Department of Physics and Astronomy, University of Texas at San Antonio, San Antonio, TX 78249, USA; Department of Physics and Astronomy, University of Texas at San Antonio, San Antonio, TX 78249, USA; Space Telescope Science Institute, Baltimore, MD 21218, USA; Center for Astrophysical Sciences, The William H. Miller III Department of Physics and Astronomy, Johns Hopkins University, Baltimore, MD 21218, USA; Solar System Exploration Division, NASA Goddard Space Flight Center, Greenbelt, MD 20771, USA; Department of Earth and Planetary Sciences, University of Texas at San Antonio, San Antonio, TX 78249, USA; Department of Physics and Astronomy, University of Texas at San Antonio, San Antonio, TX 78249, USA

**Keywords:** stardust analogs, crystallization, structural evolution, electron-beam irradiation, electron microscopy

## Abstract

The atomic microstructural evolution of circumstellar dust grains, which seed the interstellar medium, remains poorly understood. Amorphous alumina and its crystalline polymorphs, including corundum, have been found in the circumstellar shell of evolved stars. Evidence includes both astronomical observations of mid-infrared spectroscopic features and laboratory analyses of presolar grains. In this work, we show that electron fluxes can stimulate crystallization of amorphous alumina stardust analog materials using transmission electron microscopy. Crystallization experiments conducted at varying electron energies and flux conditions demonstrate a critical threshold cumulative electron dose of ∼10^24^ e^−^/m^2^ for crystallization, suggesting that the crystallization process can occur through atomic rearrangement due to electron interaction with the amorphous matrix. Throughout the crystallization process, time-resolved diffraction reveals the transition from amorphous to a transitional η-Al_2_O_3_ phase. The same transitional phase was confirmed to occur via thermal annealing at 800 °C, while annealing at 1,300 °C produced the stable crystalline phase α-Al_2_O_3_ (corundum). In both processes, the structural evolution through atomic rearrangement was characterized by quantifying the average interatomic distance between neighboring atoms using the electron pair distribution function analysis. Extrapolating to astronomical timescales, our findings suggest that electron bombardment may play a significant role in the crystallization of stardust grains, highlighting its potential importance in astrophysical environments, such as the circumstellar envelopes of planetary nebulae.

Significance statement Crystalline dust is observed in astrophysical environments where temperatures and lifetimes are insufficient for conventional thermal annealing, leaving its origin unresolved. Using laboratory astrophysics experiments that replicate electron irradiation under controlled conditions, we show that sustained electron exposure alone can crystallize amorphous alumina stardust analogs through cumulative energy deposition, producing the same transitional phases formed by high-temperature annealing. By establishing a quantitative electron-dose threshold and directly tracking atomic-scale structural evolution, this work links measurable laboratory processes to dust evolution in ionized astrophysical plasmas. These results imply that localized plasma microenvironments in planetary nebulae and related systems can drive dust crystallization on astrophysically relevant timescales, requiring a reassessment of how crystalline dust is interpreted across stellar and interstellar environments.

## Introduction

Cosmic dust influences star and planet formation, drives molecule creation in the interstellar medium (ISM), and affects the lifecycle of stars (1–3). Remarkably, dust is observed even in the earliest cosmic epochs we can detect, stretching back as far as current observational capabilities allow (4, 5). The precise way this dust interacts with its environment depends on grain size, shape, composition, and crystal structure. Until the 1990s, cosmic dust was thought to be predominantly amorphous rather than crystalline (6, 7). While the exact origin of crystalline dust grains remains unclear, the process of annealing—where heating causes amorphous materials to become more ordered—has often been proposed as a potential mechanism.

Cosmic dust is primarily composed of silicate and carbonaceous grains. However, we focus on the role of alumina for two key reasons: (i) carbon grains exhibit minimal spectroscopic features, making them challenging to study astronomically, and (ii) silicates exhibit a vast array of complex structures, complicating their analysis. In contrast, alumina is a relatively simple solid-state material that exhibits a range of distinct spectral features, making it more amenable to investigation. Thermodynamic models predict that alumina plays an important role in cosmic dust formation (8–10), confirmed by its presence in presolar grains (11). Astronomical spectra also suggest the presence of alumina grains in circumstellar (CS) dust shells (12, 13). Notably, aluminum oxides form and endure at higher temperatures than most silicates, making Al–O grains key early candidates to form dust in many astrophysical environments, and they can evolve structurally via annealing.

Alumina grains can be crystalline or amorphous and exist in a variety of polymorphs—i.e. sharing composition but differing structurally, like diamond and graphite for carbon grains. The most thermodynamically stable polymorph is α-Al_2_O_3_, commonly known as corundum. Several metastable polymorphs, identified by other Greek letters, also exist (8, 14). Observationally, gas-phase aluminum is scarce in the ISM, implying that it is largely sequestered in dust particles (7). Certain oxygen-rich evolved stars exhibit a spectral feature at ∼13 μm, which has been attributed to α-Al_2_O_3_ (15). Amorphous alumina has been invoked to explain the broad, asymmetric infrared peak at ∼11–12 μm observed around these stars (16, 17). Spectral features at ∼11, 18, 20, 28, and 32 μm have also been associated with various alumina polymorphs, as well as other species (13, 18–20). Additional description of the ∼11–12 μm feature is available in Supporting Information (SI).

In addition to astronomical studies, cosmic dust can be studied via presolar grains—i.e. meteoritic grains whose isotopic compositions indicate that they formed outside and prior to our solar system (21). Such studies of meteorites have revealed ∼250 presolar alumina grains that mostly originate from O-rich evolved stars (22–24). Laboratory analysis of two of these presolar grains has confirmed that these evolved stars can produce both amorphous and α-alumina (25). Presolar dust grains are small, rare, and difficult to extract from their parent meteorites. Consequently, experimentalists synthesize cosmic dust analogs by different methods (26, 27) for further analysis and application to understanding astronomical observations. However, many of these analogs are produced in environments that differ significantly from those in space, for example, at Earth's atmospheric pressure, producing dense glassy material (28, 29).

In this study, we examine materials with a bulk composition of alumina synthesized as smoke condensates in low-pressure conditions more closely resembling those in space. This approach allows us to analyze the initial dust grain structure and to observe how it evolves when subjected to thermal annealing or exposure to electron fluxes, providing insights into the transition from amorphous to crystalline structures.

### Electron flux environment in space and laboratory

Cosmic dust forms in relatively benign environments, such as around cool evolved stars (30) and in molecular clouds (1), where molecular gases and solids can exist. However, this dust can be dispersed from these regions into much harsher environments or the environment can itself evolve to be characterized by high temperatures, high-energy photons, shock waves, and particulate radiation. As a result, the structure of the initially formed alumina grains can evolve over time. For example, consider a low mass evolved star that is forming alumina dust. These stars undergo pulsations, generating shock waves in the dust formation zone, which impart energy to the newly formed grains (31). Some of these stars move through the galaxy with sufficient velocity to generate a bow shock (32), where the CS dust shell rams into the ISM. This CS medium–ISM interaction heats the dust and may ionize atoms, providing free electrons. As these low-mass stars evolve into planetary nebulae (PNe), the preexisting CS dust and gas are exposed to fast stellar winds and the intense radiation from a very hot stellar remnant (white dwarf, with temperatures ranging from 30,000 to >250,000 K), causing the surrounding gas to fluoresce. At this stage, the dust is subjected to both high-energy photons (UV and X-rays) and the plasma generated by these photons, which can further alter the dust.

To determine the impact of the free electrons in PNe, we estimate the unidirectional electron flux *Γ_z_* (in the *z* direction) using the electron density (*n*_e_) and electron temperature (*T*_e_), which is a measure of the average electron velocity under the Maxwellian distribution in a low-density plasma condition close to thermodynamic equilibrium. In PNe, both *T*_e_ and *n*_e_ can vary significantly: typical values for *T*_e_ are ∼10^4^ K (average kinetic energy ≈1 eV), and *n*_e_ can range between 10^2^ and 10^5^ electrons/cm^3^ (33–36). Assuming *T*_e_ = 10,000 K and *n*_e_ ≈ 10^4^ electrons/cm^3^ (based on typical values), then estimated electron flux *Γ_z_* in PNe will be ∼10^15^ e^–^/m^2^/s. However, the extreme electromagnetic radiation environment generated by the hot central stars of PNe may lead to a non-Maxwellian electron velocity distribution (37, 38). Based on similar solar system plasma environments, a so-called *κ*-distribution of electron velocity may occur. Based on (37, 38), we have calculated the electron flux for only those electrons above 1 keV for the same nebulae as above (see SI). We find the estimated electron flux *Γ_z_* (>1 keV) is ∼10^9^ e^–^/m^2^/s, 1 million times smaller than if we include all the electrons. The rationale for this 1 keV lower limit is to estimate the effect of there being a quantized energy threshold for electron-induced crystallization.

In the laboratory, we can generate an electron flux condition analogous to PNe CS environments with the column of a transmission electron microscope (TEM). The magnitude of the unidirectional electron flux can be varied by adjusting beam control parameters under parallel beam illumination mode. The notion of fluence (time-integrated flux) corresponds to the cumulative electron dose, which can be reproduced in the laboratory within a few minutes, simulating natural phenomena that occur over much longer time periods in PNe. The timescales of transformations in the lab can be used to extrapolate crystallization timescales in space.

## Results

### Crystallization under in situ electron irradiation

**Figure 1:**
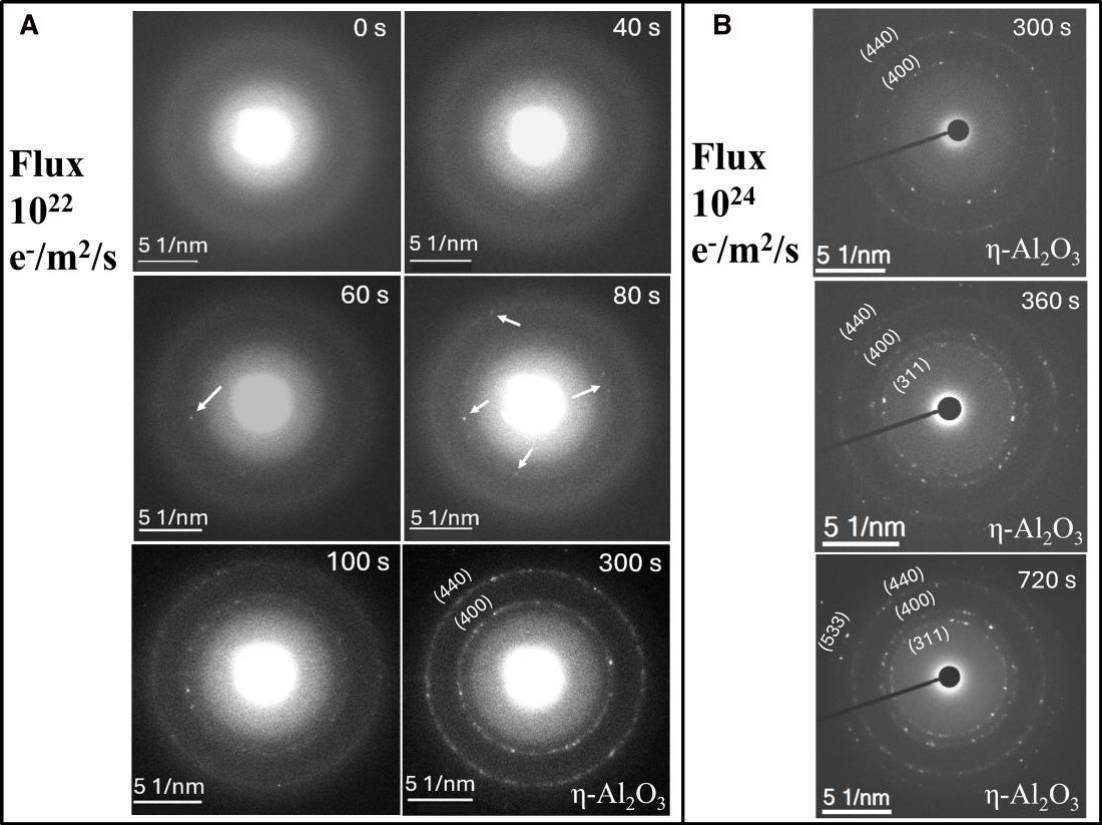
A) In situ observation of SAED patterns as a function of electron flux and exposure duration at 200 keV acquired from nearly identical regions. Debye–Scherrer rings correspond to (400) and (440) planes of cubic η-Al_2_O_3_ phase. B) Formation of additional diffraction rings after 360 and 720 s of irradiation. White arrows indicate initial spots of crystallinity.

Our stardust analog grains are highly disordered or chaotic alumina “smokes” consisting of aggregates of solid grains, each approximately tens of nanometers in size, and these grains are highly porous (39). We exposed these smoke particles to controlled electron flux in TEM by acquiring selected area electron diffraction (SAED) patterns from consistent regions of the specimen, as shown in Fig. 1A. The initial electron flux of 10^22^ electrons/m^2^/s at an incident electron energy of 200 keV was applied to a field of view of 127 nm by 127 nm (16,129 nm^2^) area at a magnification of 200,000 times. During the first 40 s of irradiation, the SAED pattern shows only diffuse halo rings characteristic of amorphous structure. At ∼60 s, initial spots indicate the formation of crystallites, denoted by white arrows in Fig. 1A, and polycrystalline rings form soon afterwards. The products of both irradiation times, 60 and 300 s, are identified as the η-Al_2_O_3_ phase. The patterns obtained at 60 s were analyzed by the electron pair distribution function (ePDF) method, while polycrystalline rings obtained at 300 s are identified by indexing their sharp and continuous rings in the SAED pattern, which is mostly attributed to many fine crystallites (10–15 nm). A parallel experiment conducted under identical electron flux conditions, but at a reduced incident electron energy of 80 keV, produced identical crystallization timescales (Fig. S1). This demonstrates that electron energy (accelerating voltage) does not significantly influence the crystallization process. A sequence of electron diffraction patterns collected with a lower electron dose of 10^21^ e^−^/m^2^/s is shown in Fig. S2. At this factor of 10 lower electron flux, no crystallization was observed until 600 s, suggesting cumulative electron dose is the key rather than the flux. These time-resolved diffraction experiments suggest that the threshold cumulative electron dose for crystallization of smoke grains is ∼10^24^ e^−^/m^2^. After irradiation, structural evolution of amorphous particles is observed in high-resolution transmission microscopy (HRTEM) images. Crystallized particles exhibit some amorphous regions, and some particles are completely crystallized. Lattice planes are observed and measured using fast Fourier transform (FFT). HRTEM micrographs and FFTs of the regions are shown in Fig. S3.

In order to test whether temperature plays a role in the crystallization process, the electron-beam irradiation was repeated at a lower temperature of 146 Kelvin degrees (−127°C) using a TEM cryo-holder using the original flux conditions (10^22^ electrons/m^2^/s at 200 keV). It was found that crystallization occurs at the same exposure time as for the experiments performed at room temperature. Figure S4 shows the sequence of SAED patterns collected at low temperature and the experimental setup of the TEM liquid nitrogen holder.

To observe the effect of higher electron flux in a comparatively small region of the sample, we increased the flux to 10^24^ e^−^/m^2^/s at an electron energy of 200 keV. Under this condition, crystallization of η-Al_2_O_3_ was identified, followed by the appearance of additional diffraction rings after 360 s. Extended irradiation for 720 s results in more pronounced η-phase formation as shown in Fig. 1B. In this case, strong reflections and partially discontinuous rings can be attributed to the larger but fewer crystallites participating in the diffraction patterns. These polycrystalline rings correspond to the (311), (400), and (440) planes of the cubic η-Al_2_O_3_ phase. Further increasing the flux to 10^25^ e^−^/m^2^/s did not induce additional crystalline phases. The parameters of electron exposure experiments are summarized in Table S1.

Another amorphous Al_2_O_3_ crystallization experiment was performed on thin films grown onto NaCl substrates using a physical process under high vacuum. The films were suspended on a TEM grid for the electron-beam irradiation (40). Compared to that study, we found that stardust analog grains require a lower electron dose (shorter exposure time) for crystallization under the same accelerating voltage of 200 keV and electron flux of 10^22^ e^−^/m^2^/s. The crystallization mechanisms of amorphous materials have been described as electron excitation, heating the medium, and knock-on (40–44). Our results show that the electron dose for crystallization is consistent regardless of incident electron energy and temperature. This suggests that crystallization can occur even at lower electron flux by reaching the threshold cumulative electron dose with a longer exposure time. Our irradiation analysis suggests that the crystallization process occurs through gradual energy deposition on amorphous smoke particles. This steady accumulation of energy promotes local atomic rearrangement within the amorphous matrix and gradually leads to crystallization.

### Structural evolution from amorphous to η-Al_2_O_3_

**Figure 2:**
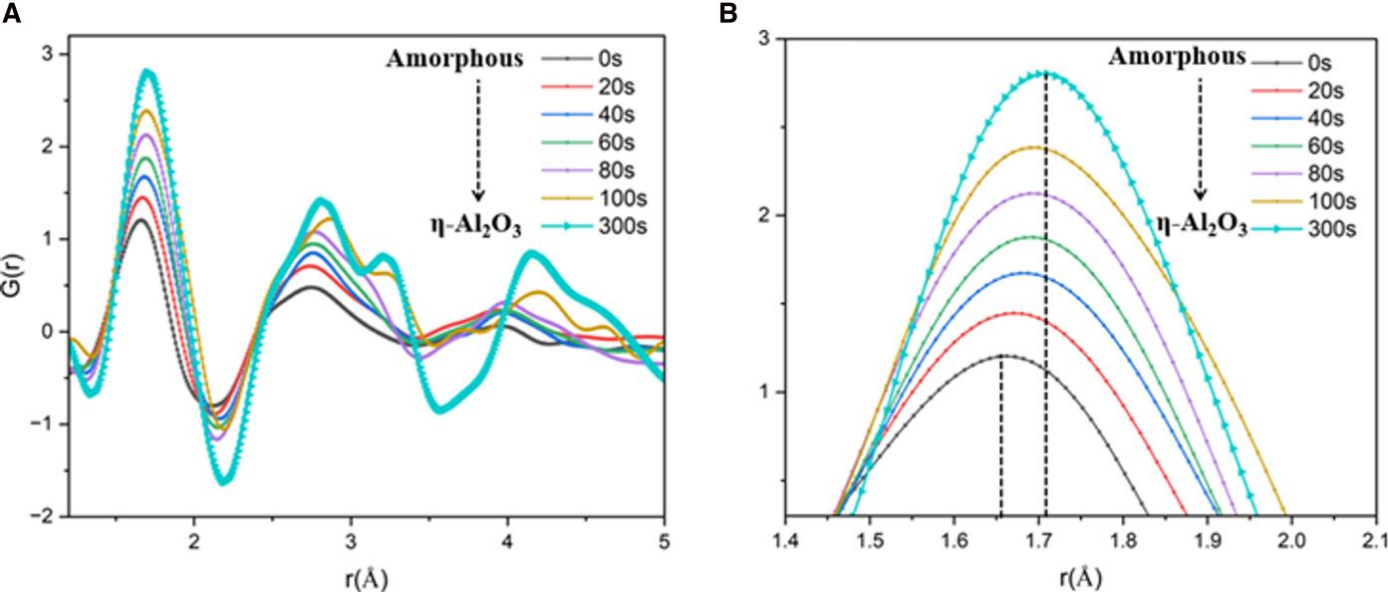
A) Evolution in pair distribution functions revealed through the analysis of SAED patterns collected from consistent specimen regions during in situ electron irradiation at 200 keV. B) The scaled-up portion of the first-neighbor distance increment as a function of irradiation time.

The local structure of amorphous materials is complex and cannot be described using simple geometric patterns. Instead, it is statistically characterized using the ePDF, denoted as G(*r*), retrieved from electron diffraction as a promising alternative to those commonly derived from neutron or synchrotron X-ray sources (45). The ePDF represents the probability of finding two neighboring atoms separated by a given distance *r* (Å); this method leverages the strong, high-resolution scattering properties of electrons. We use the Fourier transformation of the scattering intensity as a function of scattering vector magnitude to obtain the ePDF. From the collected SAED patterns under experimental conditions explained in Fig. 1A, the corresponding ePDFs were derived to investigate the structural evolution of alumina smoke particles during the amorphous-to-crystalline phase transition. Details of the ePDF data process are included in the SI. We observed sequential changes in local atomic structure throughout the transformation to the η-Al_2_O_3_ phase, characterized by a gradual increase in the first-neighbor Al–O distance with increasing electron exposure time demonstrated in Fig. 2. Specifically, the first-neighbor distance increased from 1.66 to 1.71 Å shown in Fig. 2B, reflecting local atomic rearrangement within the amorphous alumina matrix as the structure evolved toward the crystalline state. Another set of ePDF measurements under identical electron flux but at a lower electron energy of 80 keV showed a similar progressive trend in first-neighbor distance, as illustrated in Fig. S5, confirming the same mechanism for local structural changes. Both ePDFs illustrate the structural evolution toward the η-phase by gradual changes in the local atomic environment.

### Crystallization under ex situ thermal annealing

**Figure 3:**
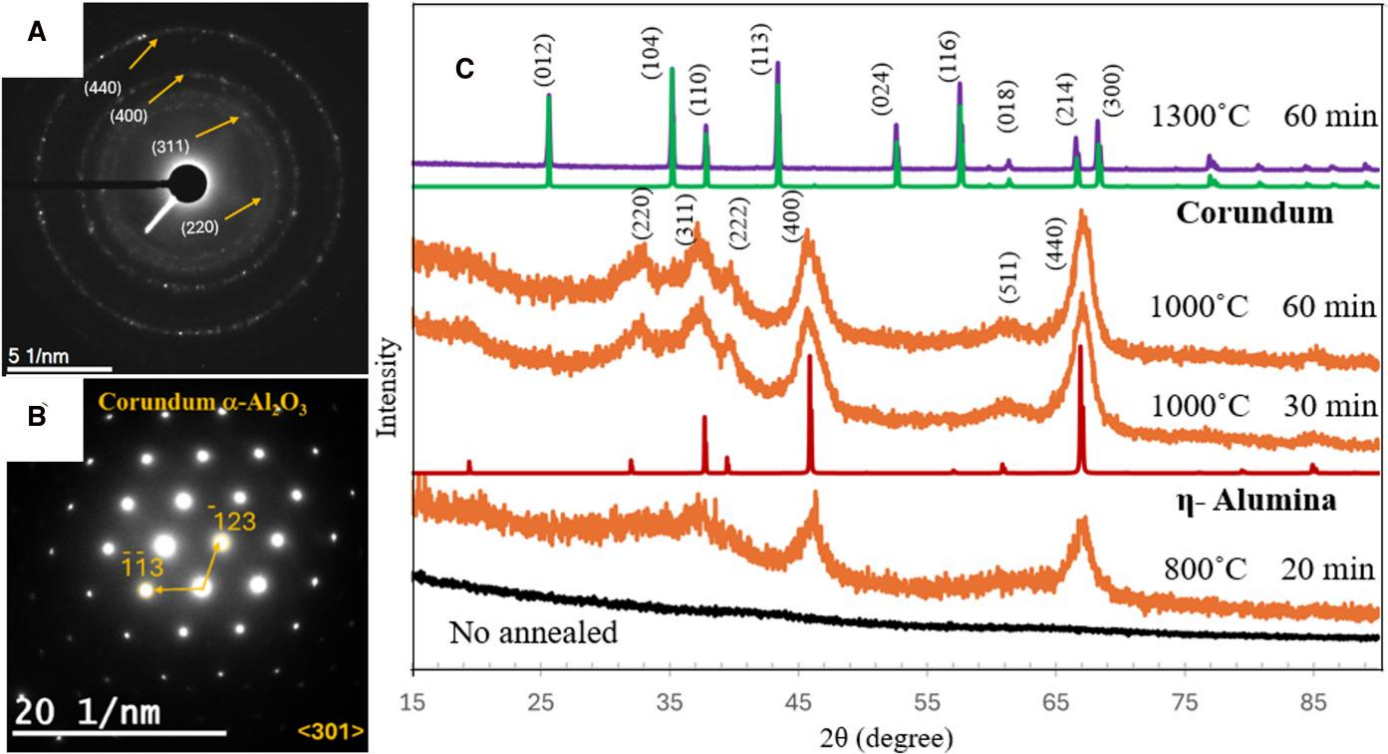
A) SAED patterns after annealing at 1,000 °C for 30 min. B) α-Al_2_O_3_ corundum phase after a second heat treatment at 1,300 °C for an hour. C) Comparative XRD analysis of both annealed alumina with reference η- and α-Al_2_O_3_ crystallographic data.

Results obtained by in situ electron-beam irradiation were further supported by the analysis of ex situ thermally annealed alumina smoke particles. Two series of annealing treatments were carried out. First, the amorphous alumina smoke condensate was heated at 400, 700, 800, and 1,000 °C for 30 min in air. The annealed samples were characterized by X-ray diffraction (XRD) and electron diffraction and indexed to the transitional phases η- (46), γ- (47), κ-, θ-, δ-, α-Al_2_O_3_, and gibbsite (hydrated alumina, γ-Al(OH)_3_; references in SI, 15–19). The analysis of the XRD and SAED patterns revealed polycrystalline formation of the η-Al_2_O_3_ phase identified by the (220), (311), (400), and (440) planes depicted in Fig. 3A and C. Radial intensity profiles of the SAED patterns are shown in (Fig. S6) and confirm identical η-phase formation in both annealing and electron irradiation experiments, indicated by perfect peak matching. The XRD profiles show that the 400 and 440 reflections have an asymmetrical profile and broadened base, which is consistent with the η-Al_2_O_3_ and not γ-Al_2_O_3_. The differences are associated with defects that induce a slight tetragonal distortion of the spinel structures, which exhibits larger distortion for γ-Al_2_O_3_ than for η-Al_2_O_3_ (48). A second sample was thermally annealed at 1,000 °C for an hour, to ensure full transformation to η-Al_2_O_3_ and subsequently annealed at 1,300 °C. This resulted in a phase transformation from η-Al_2_O_3_ to α-Al_2_O_3_, shown in Fig. 3B and C. The SAED pattern corresponds to the α-Al_2_O_3_ phase oriented along the <301> zone axis. Throughout the annealing, an increase in crystallite size was observed, and large regions of the α-phase were formed, as depicted in Fig. S7. Along with SAED analysis, XRD was performed on both first and second annealed samples and compared with the available crystallographic reference data, presented as a stacked plot in Fig. 3C. The XRD analysis confirms that all characteristic peaks from the first annealed sample correspond to cubic η-Al_2_O_3_ phase and from the second annealing correspond to α-Al_2_O_3_. These findings demonstrate that thermal annealing at 1,000 °C produces the same polycrystalline polymorphic phase as that formed during in situ electron-beam irradiation.

**Figure 4:**
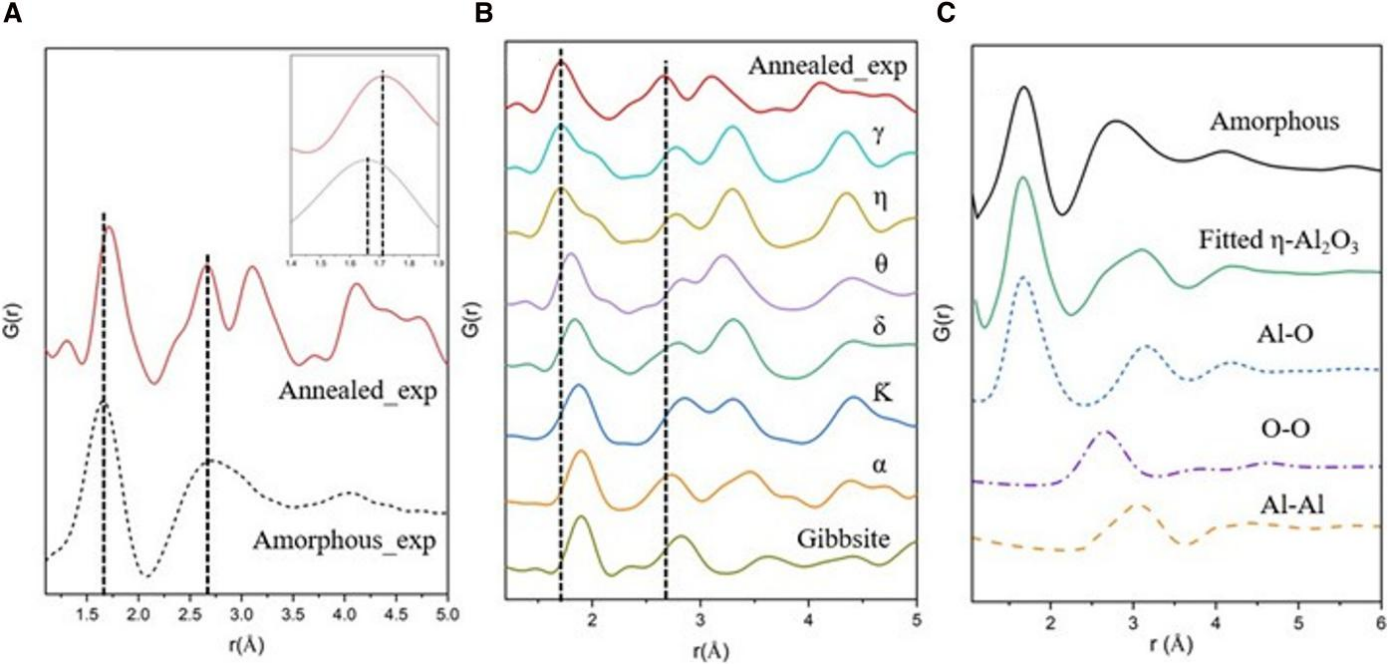
A) Experimental *G*(*r*) data measured for amorphous and thermally annealed (1,000 °C) alumina smokes from electron diffraction patterns. Inset shows the magnified section of the first peak radial shift. B) Experimentally obtained and simulated *G*(*r*) data of local structural information of different phases of aluminum oxides. Curves are normalized to give the same first shell peak height. C) Fitting of simulated η-Al_2_O_3_ phase to experimental *G*(*r*) data for amorphous alumina in the short-range order using PDFgui and partial *G*(*r*) data for Al–O, O–O, and Al–Al pairs from the γ- and η-Al_2_O_3_ fit.

In addition to indexing SAED patterns, we applied the PDF method, as described for the electron exposure experiments, with results presented in Fig. 4A. For the amorphous alumina smoke (bottom curve), the first peak appears at 1.66 Å, corresponding to the Al–O bond distance. This value aligns with simulated and experimental data reported in the literature (49) and (20 in SI), where initial peaks are observed in the range of 1.66–1.76 Å. The broad second peak, located at 2.75 Å, arises from the overlap of contributions from O–O and Al–Al bond pairs and similarly matches analogous features in the reference data. These observations indicate a consistent local atomic structure, particularly regarding Al–O and O–O/Al–Al interatomic distances. Minor discrepancies between the peak positions of amorphous alumina smoke and the data reported in the literature may be attributed to variations in sample density and preparation methods (e.g. differences in temperature or pressure). After initial annealing, the first peak slightly shifted to 1.71 Å, while the broad second peak resolved into two distinct peaks representing O–O and Al–Al bonds, and other peaks corresponding to longer distances are evident beyond the short-range order, indicating the development of medium and long-range order of η-Al_2_O_3_.

In this thermal annealing crystallization process, an increment of Al–O pair distance similar to that observed under in situ electron irradiation was found during the transition from amorphous to η-phase (Figs. 4A, inset, and 2B). The experimental *G*(*r*) data for the annealed sample were compared with simulated *G*(*r*) data for various crystalline alumina phases, as shown in Fig. 4B. In particular, the first and second peaks closely align with the simulated γ- and η-phases, suggesting that the annealed smoke material exhibits a local structure most similar to γ- and η-Al_2_O_3_. No other structures, including the thermodynamically most stable α-alumina, show local structural similarities with annealed smokes. To better understand the initial amorphous structure of the stardust analogs, a fitting was performed between experimental amorphous *G*(*r*) data and the most closely similar polymorph η-Al_2_O_3_. This fitting approach involved modification of crystalline parameters, including unit cell dimensions, atomic displacement factor, and peak broadening. Figure 4C illustrates the fittings and partial *G*(*r*) data for each atomic pair. Optimized fitting parameters are shown in Table S2, and more details are available in (50).

## Discussion

The results presented herein correlate two different causes of crystallization of alumina smokes, thermal annealing, and electron-beam irradiation. The latter is analogous to the electron fluxes in planetary nebulae. The mechanism underlying the crystallization process can be discussed based on experimental evidence. Ex situ thermal annealing crystallized the samples above 800 °C, forming the η-Al_2_O_3_ phase, while annealing at 1,300 °C crystallized the most stable alumina phase, α-Al_2_O_3_. At much lower temperatures, electron-beam irradiation caused crystallization of the η-Al_2_O_3_ phase. These two crystallization methods transfer the energy differently.

In space, condensation takes place extremely rapidly, so the initial molecular structure has neither the time nor the energy to rearrange into the α-Al_2_O_3_ form. The initial condensate therefore is more like a “frozen” gas phase, although some degree of chemical rearrangement does occur with a small degree of new bonds forming. When the sample crystallizes by thermal annealing, the Al ions become surrounded by the O cations; these small Al–O structures begin to coalesce and rearrange themselves into the transitional phases and finally the hexagonal α-Al_2_O_3_ structure. The solid starts out as very chaotic and extremely filled with defects. During the electron-beam irradiation, the process by which the Al ions become surrounded by O cations will manifest in measurements of the nearest neighbors but will produce no XRD pattern since there is no long-range order measured by the ePDF method. During this process, it is also possible to produce small clusters of Al_2_O_3_ composition. In contrast, the annealing treatments were performed in air, which provides the extra oxygen to bring the nonstoichiometric AlO solid to the transitional η-Al_2_O_3_ and the ultimate α-Al_2_O_3_ composition. Crystallization of other amorphous alumina by electron-beam irradiation occurs at longer exposure times compared with the stardust smoke analogs (41). The reason is that dense glassy alumina structures are less permeable to O diffusion, and they require a higher cumulative electron dose for crystallization.

After performing experiments at different temperatures and accelerating voltages, we conclude that the crystallization mechanism is primarily due to electron excitation, where electrons move to a higher energy level within atoms, which is determined by the electron dose. A possible secondary mechanism can be the knock-on process but only at a much higher accelerating voltage. Electron excitation produces atomic rearrangement; however, the temperature rise due to this mechanism is not significantly heating the medium, which is demonstrated by the low-temperature electron-beam irradiation.

Our results show that electron irradiation can drive the crystallization of stardust smokes in a manner similar to thermal annealing. The conditions under which the crystallization of stardust smoke analogs occurs in the TEM require an electron flux ∼10^22^ e^−^/m^2^/s for a minimum of 60 s, with full polycrystalline transformation achieved within 300 s. Therefore, a total electron exposure of ∼10^24^ e^−^/m^2^ is required to induce crystallization in amorphous alumina smokes. For a typical planetary nebula, where unidirectional electron fluxes are of the order 10^15^ e^−^/m^2^/s, this level of exposure could accumulate over ∼10^9^ s (∼30 years)—well within the typical lifetime of a planetary nebula, which spans several thousand years. However, this calculation assumes all free electrons in the nebula participate in the crystallization process. If the minimum electron energy needed to promote crystallization is >1 eV, the timescale would increase. For example, the lower flux estimated for only electrons >1 keV under a non-Maxwellian electron velocity distribution would increase the timescale by a factor of ∼1 million to ∼30 million years. Planetary nebulae are highly structured, with localized plasma microenvironments that differ substantially from nebula-averaged conditions. In particular, shocked inner rims, dense knots, and turbulent mixing or conduction fronts at hot–cool gas interfaces can sustain elevated electron densities and nonthermal or suprathermal electron populations. Therefore, it is expected that young PNe (<5,000 years old) contain regions in which electron-induced crystallization could proceed on much shorter timescales than inferred from global nebular parameters. These findings support electron bombardment as a viable mechanism for dust processing in energetic astrophysical environments. We recommend that future laboratory-based studies explore lower-energy electron fluxes than can be achieved in the TEM.

## Materials and methods

### Synthesis

The smokes were synthesized in a specialized facility that resembles a “Bunsen burner in a vacuum system” (26). In this setup, a stream of H_2_ gas containing dilute concentrations of metal-bearing molecules is introduced into a furnace. Within the furnace, the gas mixture combines with an oxidizer, producing a ∼1,500 K flame that can interact with a metal sample housed in a graphite crucible. The metal's vapor pressure is controlled by adjusting the furnace temperature, typically set at ∼750 K, though it has been pushed as high as 1,200 K. The gas residence time within the furnace ranges from 5 to 10 ms, and each run yields ∼0.5 g of smoke material. Depending on the synthesis conditions, the smokes are formed by metastable amorphous alumina.

### Powder X-ray diffraction

Laboratory powder XRD patterns of alumina were collected using a Panalytical Empyrean X-ray diffractometer at room temperature, which uses Cu Kα radiation with a wavelength of 1.54056 Å. To prepare samples for XRD analysis, small amounts of as-synthesized and annealed alumina powder were uniformly dispersed on the sample holder.

### TEM sample preparation and electron-beam irradiation conditions

For electron microscopy analysis, the powdery smoke samples were mixed into isopropanol by sonicating for 20 min to get a homogeneous mixture. Aliquots of 3–5 µL of the mixture were drop-cast using a pipette onto a commercial TEM grid, LC300-Cu-100 (Lacey carbon–copper grid), mesh (300) thin carbon film (thickness ≈5 nm) and then left until dry. For as-synthesized and annealed smoke samples, SAED patterns were collected with a 16-bit CMOS camera in a JEOL 2010F microscope. Electron-beam irradiation experiments were performed using a JEOL coldFEG JEM-F200 microscope equipped with a 16-bit Rio Camera CMOS under different electron flux conditions and operated at two accelerating voltages, 80 and 200 kV.

### ePDF methodology

The PDF is defined as the Fourier transformation *G*(*r*) of the *Q*-space total scattering data and represents the weighted probability of finding two neighboring atoms separated by a given distance *r*. Scattering intensity, *I*(*Q*) is a continuous function of the scattering vector *Q*, scattering angle *θ* and wavelength *λ*. A sine Fourier transform over the structure function *S*(*Q*) between the minimum and maximum measured values of *Q* directly derives the PDF, *G*(*r*).


(1) $$G(r)=\frac{2}{\pi }\int_{\;Q\min}^{Q\max}Q[S(Q)-1]\sin\;(Qr)\;\;dQ\;$$

The *G*(*r*) function carries a wealth of important structural information, and it is the most used real-space function obtained directly from the diffraction data.

## Supplementary Material

pgag095_Supplementary_Data

## Data Availability

The data underlying this article is available in the article and in its Supplementary material.

## References

[1] Draine BT . 2003. Interstellar dust grains. Annu Rev Astron Astrophys. 41:241–289.

[2] Krishna-Swamy KS . Dust in the universe: similarities and differences. World Scientific, 2005.

[3] Krugel E . An introduction to the physics of interstellar dust. CRC Press, 2007.

[4] Dwek E, Cherchneff I. 2011. The origin of dust in the early universe: probing the star formation history of galaxies by their dust content. Astrophys J. 727(2):63.

[5] Witstok J, Jones GC, Maiolino R, Smit R, Schneider R. 2023. An empirical study of dust properties at the earliest epochs. Mon Not R Astron Soc. 523(2):3119–3132.

[6] Draine BT, Lee HM. 1984. Optical properties of interstellar graphite and silicate grains. Astrophys J. 285:89–108.

[7] Whittet DC . Dust in the galactic environment. CRC Press, 2018.

[8] Grossman L . 1972. Condensation in the primitive solar nebula. Geochim Cosmochim Acta. 36:597–619.

[9] Lattimer JM, Schramm DN, Grossman L. 1978. Condensation in supernova ejecta and isotopic anomalies in meteorites. Astrophys J. 219:230–249.

[10] Dominik C, Sedlmayr E, Gail HP. 1993. Dust formation in stellar winds. VI. Moment equations for the formation of heterogeneous and core-mantle grains. Astron Astrophys. 277:578.

[11] Takigawa A, et al 2014. Morphology and crystal structures of solar and presolar Al_2_O_3_ in unequilibrated ordinary chondrites. Geochim Cosmochim Acta. 124:309–327.

[12] DePew K, Speck A, Dijkstra C. 2006. Astromineralogy of the 13 μm feature in the spectra of oxygen-rich asymptotic giant branch stars. I. Corundum and spinel. Astrophys J. 640(2):971–981.

[13] Sargent BA . 2018. Alumina polymorphism in the circumstellar dust shells of asymptotic giant branch stars. Astrophys J Lett. 866(1):L1.

[14] Levin I, Brandon D. 1998. Metastable alumina polymorphs: crystal structures and transition sequences. J Am Ceram Soc. 81(8):1995–2012.

[15] Sloan GC, Kraemer KE, Goebel JH, Price SD. 2003. Guilt by association: the 13 micron dust emission feature and its correlation to other gas and dust features. Astrophys J. 594(1):483–495.

[16] Speck AK, Barlow MJ, Sylvester RJ, Hofmeister AM. 2000. Dust features in the 10-μm infrared spectra of oxygen-rich evolved stars. Astron Astrophys Suppl Ser. 146(3):437–464.

[17] Onaka T, De Jong T, Willems FJ. 1989. A study of M Mira variables based on IRAS LRS observations. I. Dust formation in the circumstellar shell. Astron Astrophys. 218:169–179.

[18] Molster FJ, Waters LBFM, Tielens AGGM. 2002. Crystalline silicate dust around evolved stars-II. The crystalline silicate complexes. Astron Astrophys. 382(1):222–240.

[19] Tamanai A, et al 2009. Morphological effects on IR band profiles-experimental spectroscopic analysis with application to observed spectra of oxygen-rich AGB stars. Astron Astrophys. 501(1):251–267.

[20] Niyogi SG, Speck AK, Onaka T. 2011. A temporal study of the oxygen-rich pulsating variable asymptotic giant branch star, T Cep: investigation on dust formation and dust properties. Astrophys J. 733(2):93.

[21] Clayton DD, Nittler LR. 2004. Astrophysics with presolar stardust. Annu Rev Astron Astrophys. 42(1):39–78.

[22] Nguyen AN, et al 2007. Characterization of presolar silicate and oxide grains in primitive carbonaceous chondrites. Astrophys J. 656(2):1223–1240.

[23] Huss GR, Fahey AJ, Gallino R, Wasserburg GJ. 1994. Oxygen isotopes in circumstellar Al203 grains from meteorites and stellar nucleosynthesis. Astrophys J. 430:L81–L84.

[24] Bose M, Floss C, Stadermann FJ. 2010. An investigation into the origin of Fe-rich presolar silicates in Acfer 094. Astrophys J. 714(2):1624.

[25] Stroud RM, Nittler LR, Alexander CMOD. 2004. Polymorphism in presolar Al_2_O_3_ grains from asymptotic giant branch stars. Science. 305(5689):1455–1457. 15353800 10.1126/science.1101099

[26] Nuth JA III, Hallenbeck SL, Rietmeijer FJ. 2000. Laboratory studies of silicate smokes: analog studies of circumstellar materials. J Geophys Res Space Phys. 105(A5):10387–10396.

[27] Vidali G, Roser JE, Manicó G, Pirronello V. 2004. Laboratory studies of formation of molecules on dust grain analogues under ISM conditions. J Geophys Res Planets. 109(E7):E07S14.

[28] Speck AK, Whittington AG, Hofmeister AM. 2011. Disordered silicates in space: a study of laboratory spectra of “amorphous” silicates. Astrophys J. 740(2):93.

[29] Dorschner J . Properties of interstellar dust. In: Gustafson BAS, Hanner MS, editors. Physics, chemistry, and dynamics of interplanetary dust. Astronomical Society of the Pacific Conference Series; Proceedings of the 150th colloquium of the International Astronomical Union held in Gainesville; Florida; USA; 14–18 August 1995; Astronomical Society of the Pacific (ASP 104); c1996. Vol. 104. San Francisco, 1996. p. 487.

[30] Gail HP, Sedlmayr E. Physics and chemistry of circumstellar dust shells. Vol. 52, Cambridge University Press, 2014.

[31] Gobrecht D, Cherchneff I, Sarangi A, Plane JM, Bromley ST. 2016. Dust formation in the oxygen-rich AGB star IK Tauri. Astron Astrophys. 585:A6.

[32] Martin DC, et al 2007. A turbulent wake as a tracer of 30,000 years of Mira's mass loss history. Nature. 448(7155):780–783. 17700694 10.1038/nature06003

[33] Osterbrock DE . 1960. Electron densities in planetary nebulae. Astrophys J. 131:541.

[34] Toalá JA, Arthur SJ. 2016. Formation and X-ray emission from hot bubbles in planetary nebulae-II. Hot bubble X-ray emission. Mon Not R Astron Soc. 463:4438–4458.

[35] Stanghellini L, Kaler JB. 1989. Electron densities in planetary nebulae. Astrophys J. 343:811–827.

[36] Zhang Y, et al 2004. Electron temperatures and densities of planetary nebulae determined from the nebular hydrogen recombination spectrum and temperature and density variations. Mon Not R Astron Soc. 351(3):935–955.

[37] Nicholls DC, Dopita MA, Sutherland RS. 2012. Resolving the electron temperature discrepancies in H II regions and planetary nebulae: κ-distributed electrons. Astrophys J. 752(2):148.

[38] Zhang Y, Zhang B, Liu XW. 2016. On the nonthermal κ-distributed electrons in planetary nebulae and H II regions: the κ index and its correlations with other nebular properties. Astrophys J. 817(1):68.

[39] Nuth JA, Hecht JH. 1990. Signatures of aging silicate dust. Astrophys Space Sci. 163:79–94.

[40] Nakamura R, Ishimaru M, Yasuda H, Nakajima H. 2013. Atomic rearrangements in amorphous Al_2_O_3_ under electron-beam irradiation. J Appl Phys. 113(6):064312.

[41] Sigmund P . Particle penetration and radiation effects: general aspects and stopping of swift point charges. Springer Berlin Heidelberg, Berlin, Heidelberg, 2006.

[42] Gerasimova K, Aliev VS, Krivyakin GK, Voronkovskii VA. 2020. Comparative study of electron-beam crystallization of amorphous hafnium oxides HfO_2_ and HfO_x_ (x= 1.82). SN Appl Sci. 2(7):1273.

[43] Murray J, Song K, Huebner W, O'Keefe M. 2012. Electron beam induced crystallization of sputter deposited amorphous alumina thin films. Mater Lett. 74:12–15.

[44] Corbett JW . Electron radiation damage in semiconductors and metals. Academic, New York, 1966.

[45] Billinge SJ, Kanatzidis MG. 2004. Beyond crystallography: the study of disorder, nanocrystallinity and crystallographically challenged materials with pair distribution functions. Chem Commun. 7:749–760. 10.1039/b309577k 15045050

[46] Shirasuka K, Yanagida H, Yamaguchi G, Kyokaishi Y. 1976. The preparation of eta alumina and its structure. J Ceram Assoc Jpn. 84(976):610–613.

[47] Smrčok L, Langer V, Křesťan J. 2006. γ-Alumina: a single-crystal X-ray diffraction study. Acta Crystallogr C. 62:i83–i84. 16954611 10.1107/S0108270106026850

[48] Pecharromán C, González-Carreño T, Iglesias JE. 1996. The infrared dielectric properties of η–Al2O3. J Mater Res. 11(1):127–133.

[49] Gutiérrez G, Johansson B. 2002. Molecular dynamics study of structural properties of amorphous Al_2_O_3_. Phys Rev B. 65(10):104202.

[50] Shohan RA . 2025. Structural transformation and physical properties of oxide materials by using in-situ transmission electron microscopy (Order No. 32396210). Available from Dissertations & Theses @ University of Texas—San Antonio; ProQuest One Academic; ProQuest One Academic (3281674968).

